# A qualitative study of the experiences and expectations of women receiving in-patient postnatal care in one English maternity unit

**DOI:** 10.1186/1471-2393-10-70

**Published:** 2010-10-27

**Authors:** Sarah Beake, Val Rose, Debra Bick, Annette Weavers, Julie Wray

**Affiliations:** 1 Kings College, London, Florence Nightingale School of Nursing and Midwifery, London UK; 2 Royal Berkshire NHS Foundation Trust, Reading, UK; 3 The University of Salford, School of Nursing and Midwifery, Manchester, UK

## Abstract

**Background:**

Studies consistently highlight in-patient postnatal care as the area of maternity care women are least satisfied with. As part of a quality improvement study to promote a continuum of care from the birthing room to discharge home from hospital, we explored women's expectations and experiences of current in-patient care.

**Methods:**

For this part of the study, qualitative data from semi-structured interviews were transcribed and analysed using content analyses to identify issues and concepts. Women were recruited from two postnatal wards in one large maternity unit in the South of England, with around 6,000 births a year.

**Results:**

Twenty women, who had a vaginal or caesarean birth, were interviewed on the postnatal ward. Identified themes included; the impact of the ward environment; the impact of the attitude of staff; quality and level of support for breastfeeding; unmet information needs; and women's low expectations of hospital based postnatal care. Findings informed revision to the content and planning of in-patient postnatal care, results of which will be reported elsewhere.

**Conclusions:**

Women's responses highlighted several areas where changes could be implemented. Staff should be aware that how they inter-act with women could make a difference to care as a positive or negative experience. The lack of support and inconsistent advice on breastfeeding highlights that units need to consider how individual staff communicate information to women. Units need to address how and when information on practical aspects of infant care is provided if women and their partners are to feel confident on the woman's transfer home from hospital.

## Background

Around 650,000 women gave birth in England during 2007 - 2008 [1], the majority of births taking place in hospital. Giving birth is for most women in the United Kingdom (UK) their first experience of being admitted to hospital and although in general they may be content with their experience of care during labour, their experience of hospital care after giving birth has been consistently evaluated as poor [2-5]. This is not just a UK phenomenon, with a growing body of evidence that postnatal hospital care is also reported negatively in other developed countries [6-10].

There is often a mismatch between what women expect to receive from their maternity care and the level of service provided [11] with a perceived lack of support from staff in the postnatal period, in particular concerning infant feeding and practical aspects of infant care such as bathing and changing the baby [3,6,7,9]. Women perceive that staff are often rushed and too busy on postnatal wards to provide the care they feel they require, in particular to meet their emotional needs [3,6] and it has been suggested that there is a need to improve the communication and listening skills of staff [6]. The Healthcare Commission [5] (now known as the Care Quality Commission) recommended that women require information and support during the early postnatal period in order for them to 'bond with their baby, become skilful in techniques of feeding and grow in confidence as parents' (p9).

During the last 20 years in the UK, the length of time most women spend in hospital after giving birth has declined, despite increases in interventions during labour and birth, including a rise in the number of caesarean births [1], the increase in reported adverse obstetric events [12] and the poorer general health of women who become pregnant [13]. In the UK the average postnatal stay in hospital for a normal vaginal birth is now just under 1.5 days, for an assisted vaginal birth two days and for a caesarean section approximately 3.5 days [5]. The first few postnatal days are a crucial time for women to obtain information and support to enable them to establish breastfeeding, develop in confidence as a mother and prepare them for their transfer home [5]. The importance of effective care during the inpatient period has been highlighted, not only due to the increase in interventions leading to higher maternal physical and psychological morbidity, but also as a consequence of pressures on resources reducing hospital turnover intervals and reduction in the number of midwife contacts a woman may receive once home [3]. Changes in the staffing skill-mix are also taking place across the UK, with more maternity support workers and general nurses being employed by hospitals to work in the postnatal area. In some parts of the UK, a lack of midwives has resulted in more women receiving care in hospital and at home from maternity support workers who have a range of different tasks and responsibilities delegated to them [14]. Findings from a recent national survey suggests a changing pattern of home based visits, with more women receiving contacts up to 28 days of birth, although the longer duration of contact was not associated with an increase in the number of contacts [15].

Midwifery postnatal care has historically focused on routine observations and examinations to monitor physical recovery from birth. This proscriptive care was initially introduced to identify the onset of haemorrhage and sepsis post-birth, which were associated with high maternal mortality in the UK at the turn of the 20^th ^century [16]. However, the emphasis on monitoring physical recovery has meant that there has been little consideration given to women's emotional needs after giving birth. Recent drivers to promote more efficient use of finite healthcare resources, resulting in the emphasis on shorter-inpatient stay, has also appeared to move the focus away from provision of parent education on postnatal wards and demonstration of practical aspects of infant care. Large observational studies published since the 1990's in the UK and elsewhere identified widespread and persistent maternal morbidity which was not reported by women or identified by health care staff [17-19]. National guidance for National Health Service (NHS) care in England and Wales now advises that women should be asked about their emotional well being at each postnatal contact and that they have an initial assessment of needs and individualised plan of care [20,21].

Supporting women to breastfeed is an important part of postnatal care and a public health priority. Despite this and the level of robust evidence available on the shorter and longer-term benefits of breastfeeding for the woman and her infant [22], rates in the UK remain among the lowest in Europe. The Infant Feeding Survey 2005 showed an overall breastfeeding uptake of 76% in the UK, an increase on previous years, however breastfeeding rates remain highest among women from managerial and professional occupations, those aged over 30 and among first time mothers [23]. In the 2005 survey breastfeeding rates had decreased to less than half within six weeks of the birth with only 21% of women exclusively breastfeeding. At six months after the birth, the number of women exclusively breastfeeding was negligible (< 1%) [23]. Increasing breastfeeding rates in the UK is currently a national public service agreement initiative (PSA). This commissioning guidance aims to assist health service commissioners and primary care trusts responsible for services in the secondary care sector to provide coherent services to promote breastfeeding and reduce inequalities, as set out in the policy report 'Healthy Lives, Brighter Futures' [24]. The PSA target is to increase the prevalence of exclusive breastfeeding at six to eight weeks and the percentage of women who breastfeed at 7 to 9 months along with appropriate amounts and types of solid food [25].

In addition to improving child health outcomes, there has been an increased UK policy focus on the safety and quality of maternity care, with a rise in the number of 'adverse events' reported in the maternity services, prompted by more effective reporting systems and willingness of staff to report these [26]. 'Towards Better Births' [5] which presented findings of a review of the maternity services in England, reported that inadequate staffing levels, failure to communicate with women and lack of in-service training for staff were leading to poor outcomes of care. A number of recommendations were made for postnatal care. These included that women should not be transferred from hospital too early, particularly after a caesarean section, that length of stay in hospital should be negotiated and take account of the health and wellbeing of the woman and her baby and the level of support available to the woman after transfer. The review also recommended that hospitals needed to establish more highly rated postnatal care packages, including increasing the number of contacts in the home and addressing issues such as infant crying and skin care.

This paper presents data from 20 women who were interviewed on two postnatal wards as part of a quality improvement study, informed by a model of continuous quality improvement (CQI). The study aimed to identify where routine systems and processes of care in hospital following birth could be revised to enhance women's experiences, promote care tailored to need and promote a continuum of care from labour ward to transfer home. Several phases were completed prior to implementing changes in routine practice. These included the development and review of a process map of several women's 'journeys' through the hospital immediately following birth until their discharge home which provided a useful schema of where bottle-necks in the system existed and why staff in the relevant clinical areas considered that this might be the case. Mapping is a commonly used quality improvement method to enhance the safety and quality of health care [27], although there is little published evidence of its use in maternity care. Interviews and focus groups were also held with hospital and community-based midwives, senior midwives and obstetricians and clinical managers to ascertain their views on how in-patient postnatal care could be improved.

In preparation to improve care in line with the needs of women using the service, we aimed to explore their experiences and expectations of current in-patient postnatal care. Obtaining evidence of the views of women was essential to ensure these were reflected in revisions to service delivery. Details of the changes to systems and processes of care identified in our preliminary work and outcomes following implementation of changes to care will be published elsewhere. The study was funded by a grant from the Burdett Trust for Nursing.

## Methods

The overall study was informed by a mixed methods approach to ensure strategies to inform improvements in care systems and processes were relevant and timely, reflected the perspectives of women, clinicians and managers and were pragmatic for implementation in the clinical areas involved. This included qualitative approaches, for example, to explore women's views (as reported here), focus groups and interviews with staff, and a quantitative approach namely a pre and post-intervention survey of women at 10 days and 3 months after giving birth and a survey of staff views of revisions to care made (to be reported elsewhere).

Recruitment of women took place on the two postnatal wards in a District General Hospital in the South of England with around 6,000 births a year. At the time the study was conducted, 28% of women had a caesarean birth, 58% a spontaneous vaginal birth, and 14% a vaginal instrumental birth. Around 95% of women booked with the unit gave birth in hospital, with a 5% home birth rate. One postnatal ward was part of an integrated midwifery-led unit for low risk women and their babies and the other admitted high risk postnatal women and their babies, and women whose babies required transitional care.

Women who agreed to take part were interviewed in a room off the main ward areas by the research midwife (VR) at a pre-arranged time prior to their discharge home. Interviews were semi-structured and included questions for women on their views of whether in-patient postnatal care had met their expectations, their overall experiences of care, if they regarded that their emotional and physical health needs had been met, and if any support needs for practical aspects of infant care, including infant feeding had been addressed. Questions which reflected national guideline recommendations for postnatal care within the first 24 hours [21] were also asked (for example, if women had an opportunity to talk about their birth and were offered advice on signs and symptoms of potentially adverse health problems, for example sepsis or pre-eclampsia).

### Data analysis

The interviews were transcribed using content analysis [28]. As a first step, two researchers (DB and SB) independently examined the interview transcripts to identify issues and concepts apparent in the data. Units of meaning in each sentence or paragraph were given descriptive codes or labels. Following these initial steps, the two researchers discussed areas of agreement and discrepancy and further refined the coding scheme. As the next step, the codes were grouped into more abstract categories with various dimensions and overall themes or core categories identified. An analytical framework was then developed and applied across all the interviews as a means of re-organising the data at a greater level of abstraction. The final step involved writing up memos on each theme which grouped views, experiences and quotes according to their appropriate thematic reference and helped the researchers further refine their explanations, draw associations and understand the weight and range of certain findings. The sample size was determined when saturation of information was achieved and all interviews were tape recorded with the woman's permission. The interview recordings were transcribed by the research team administrator. As data from the women were confidential, a code was allocated to each woman to protect her anonymity.

### Ethics approval

Ethical approval was obtained via the National Research Ethics Committee (reference number 07/H0505/124). All women who agreed to take part were asked to provide written consent after reading an information leaflet about the study offered to women on the postnatal ward by the research midwife.

## Findings

Twenty women whose ages ranged from 23 to 39 were recruited from the two postnatal wards, a number considered appropriate to provide sufficient data to meet the objectives of this stage of the study (data saturation). Most were white European; one woman was Afro-Caribbean and one was Chinese. A higher proportion of women had given birth to their first baby (13/20), the number of previous births among the other seven women ranging from two to four. Over half of the women who agreed to take part had had an emergency caesarean section birth (12/20), three women had a planned caesarean birth, two women a spontaneous vaginal birth and three women an assisted vaginal birth.

The main themes that emerged from the interviews were; the impact of the environment of the postnatal ward on birth recovery; the attitude of clinical and non clinical staff; the level of support for breastfeeding; unmet information needs; and women's expectations. Data on mode of birth, age and parity of each woman are presented in Table 1

**Table 1:** Mode of birth, age and parity of women interviewed

Study Code	Previous births	Mode of birth	Age
A5	3	SVD	31

A6	0	Elective CS	26

A7	0	Emergency CS	35

B1	1	Elective CS	33

B2	0	Emergency CS	30

B3	0	Forceps	28

B4	0	Emergency CS	35

B5	0	Emergency CS	34

C1	0	Emergency CS	39

C2	0	SVD	38

C3	0	Ventouse	32

C5	3	Emergency CS	38

C6	0	Emergency CS	23

C8	2	Emergency CS	25

C9	2	Emergency CS	36

C10	0	Forceps	39

C11	0	Emergency CS	35

D1	1	Elective CS	29

D2	3	Emergency CS	37

D3	0	Emergency CS	35

### The impact of the environment of the postnatal ward on birth recovery

Ward routines, such as the lights being switched on early in the morning and kept on until late at night, were something that women found difficult to comprehend. It led them to perceive that in some cases routines were more often in place for the convenience of the staff than for their own needs. Getting enough rest in the hours following birth which may have been preceded by a long period of labour, and subsequent physical pain, for example from perineal or abdominal wounds, is difficult with the demands of a newborn baby. For the women interviewed it was apparent that their attempts to rest were made worse by hospital routines:

'The lights are turned on at half six in the morning' (A5)

or conversely kept on until late at night:

'The thing is it might be silly, especially because of the baby: the lights are kept on until very late......... so I wondered at night why the lights have to be kept on so brightly' (B5)

It was not only that lights were switched on early in the morning. Procedures such as removal of a urinary catheter were also undertaken first thing in the morning. One woman said:

'I would prefer not to have been woken up at six in the morning to have my catheter removed' (D1)

Issues with the long day did mean that women were appreciative of the provision of a 'quiet time' during the day when the ward was closed to visitors other than their partners so that they could catch up on much needed rest:

'I do like the two hour quiet, no visitors, one until three. I think that's a really important thing' (B2)

In terms of ward layout, some of the women interviewed were happy to share a room, while some preferred to be on their own as they were worried that they were disturbing other women on the ward. It was not clear if the women interviewed had been offered a choice of a separate room on the ward, as the hospital did provide amenity rooms. Single rooms were also offered to women who were ill following the birth or if their baby was on the neonatal unit. Women who had experienced a stillbirth or neonatal death would also be offered a room of their own:

'I don't think I would want to be on my own. It's quite nice to talk to other people' (A6)

'I think probably if I might have had a separate room I may not have been so concerned about her crying last night because she was keeping others awake' (C2)

It was perhaps not unexpected that hospital food was another area of discontent, although this was not in terms of the quality of the food but in terms of quantity. As women recovering from labour and giving birth, the provision of good nutrition is important for supporting recovery and commencement of breastfeeding. One woman said:

'If anything with the food I have found that I have been a little bit hungry....... So I had to get my partner to bring fresh fruit' (C1)

### Staff attitude on the postnatal ward

For the most part staff, the midwives, nursery nurses (a person qualified in non-clinical care of babies and young children), maternity care assistants and house keepers, who women came into contact with on the postnatal wards, were described very positively. Women commonly used words such as friendly, helpful and polite and articulated that the care they gave was excellent:

'the nursery nurses who I think have been fabulous, I would be totally lost if they weren't here, they have really helped me' (C11)

'I think people are just really friendly and helpful, and nothing seems to be, you know, if you ask nothing is too much trouble' (B1)

Women acknowledged that all staff were busy. There were some comments made about call bells not being answered, or that staff were slow to answer when women called for them. As a consequence women felt guilty about bothering staff, as the quote below illustrates:

'Because they are so busy you don't want every time you need to change the sheet or something you don't want to buzz and say can you get me another sheet' (A7)

A small minority of staff were described negatively, in most cases following a particular individual encounter where the women felt that the member of staff had spoken in a 'sharp' or 'abrupt' manner:

'The vast majority of staff are very approachable and very nice..... There was one woman last night who was a bit kind of officious but she has really been the only one' (D1)

### The level of support for breastfeeding

The quality and depth of breastfeeding advice varied depending on which individual member of the health care team the woman had seen. In some cases, it was reported as excellent, with women highly appreciative of the individual time some staff offered them:

'I have been impressed by the number of hours, the amount of bedside time that the staff working here are able to provide rather than just saying I will come and spend a few minutes trying to help you .... People have been prepared to come and spend an hour solid, not running in and out, but just spend an hour full stop helping you' (B2)

Of particular note was that the nursery nurses were identified as providing excellent support with breast feeding, perhaps as they appeared less rushed and had more time to help:

'All the help with the breastfeeding, because all the nursery nurses, all the ladies in their purple t-shirts have been fantastic' (A7)

However there were some staff who women perceived as being unhelpful, leaving them with the impression that they did not want to promote breastfeeding:

'she was not keen to help me and she would say you don't need to breastfeed if you don't want you can use the bottle' (C6)

There was also frustration about requests for help not being followed up by staff, as one woman described in her experience:

'If I ask 'can you get me a bottle of milk' or' I need help with feeding the baby' they sort of go away and don't come back for another hour, two hours later. And by that time it's like I tried to breastfeed him at first and asked for help because he wouldn't take it, he was taking a long time, so I just had to bottle feed him' (B3)

Although the National Institute of Health and Clinical Excellence (NICE) [21] and the World Health Organization (WHO)/United Nations Children's Fund (UNICEF) Baby Friendly Hospital Initiative [29] recommend 24 hour rooming-in, some women did appreciate having their baby taken to be looked after by the midwives' overnight so that they could get some rest. One woman reported that she had not expected this 'service' on the postnatal ward:

'I didn't expect to have the nursery service at night. And that was lovely at night, my first evening, just to be able to know someone was looking after her, bringing her back for feeds and that was just fantastic' (C3)

### Unmet information needs

Women were asked about the sort of information they felt they needed on discharge home from hospital. For many women it was information that would help them to develop their confidence in 'basic' practical aspects of care of their baby such as nappy changing and bathing the baby.

For first-time mothers, a range of unmet information needs were raised, as illustrated in the following quote:

'Everyone tells you how to breastfeed, but it's like how often do you breastfeed and do you pick up the child up every time she cries. If you are a first time mum this is all new. And how often do you change her nappy? Or are you checking all the time?' (B2)

For many women giving birth was the first time they had been admitted to hospital. As a result they were unfamiliar with the ward environment and hospital routines. Inadequate orientation and explanation by staff often increased women's anxiety, especially if staff did not explain routines to them or show them where to find what they needed on the ward, as illustrated by the comments below:

'I didn't know where to get milk or the bathroom or anything because nobody really told me' (A5)

'There is nothing bad but for me I think I don't know really what is going on or what should happen, or what I should be doing' (C11)

'I would have liked someone to come in on my first day and say right this is what happens, you are here for three days four days and this is what we will be doing this day' (C11)

For the women who had given birth by caesarean section, it was clear that they felt they needed practical advice and information on what they could or could not do once they were home. Women spoke of needing information on how often they should wash their abdominal wound area, how to take care of their wound including use of dressings, and if (and when) they could undertake practical tasks such as walking up stairs and driving. Women also wanted to know who to contact if something went 'wrong' following discharge from hospital:

'Your top do's and don'ts sort of thing, if somebody has had a 'C' section and going home..... when do my stitches come out?' (B2)

This comment reflected that there was little advice offered to women in general on aspects of recovery from birth as recommended in national guidance (NICE 2006) [21].

### Women's expectations

All of the women were asked about their expectations of hospital-based postnatal care before they were admitted to hospital. Their responses were varied, with some women feeling that their experience was better than they had expected, as referred to in the following quote:

'I had reasonably high expectations and I feel that they have been met' (B1)

Although it was disappointing to note that some women had not expected a very high level of care from the postnatal ward, it was encouraging that they were subsequently surprised when their expectations were exceeded as the following illustrates:

'I didn't come in with high expectations, I know how busy everyone is, and my midwife has been telling me there is a massive shortage so you know I was just quite pleased that there was a bed and the midwife was nice' (A5)

## Discussion

This study presents the first in-depth views of women in one UK maternity unit obtained at the time they were receiving in-patient postnatal care. Interviews with women were one of several approaches used by the study team to inform how the content, systems and processes of in-patient postnatal care could be revised to better reflect women's needs. Although the focus of the quality improvement initiative was the postnatal ward, it soon became clear that consideration would also have to be given to care processes during the antenatal and intra-partum periods, if transfer from the delivery suite to the postnatal ward and home were to be 'seamless'. The findings have reiterated that although many women were happy with the care they received in hospital, some were disappointed with aspects of their care and others went into hospital with low expectations of their admission to a postnatal ward. Most women were able to identify areas where they felt improvements could be made. Analyses revealed issues about the physical environment of the ward, the approaches and attitude of staff, breastfeeding support, level of practical support and guidance on infant care and how to take care of their health. It is hoped that this work will influence others to consider how to ensure women's views of in-patient postnatal care are captured, given the recognition that user engagement and feedback is essential if high quality patient care is to be achieved [30].

There is a dearth of in-depth information on UK women's views of their in-patient postnatal care. This is despite the provision of statutory care since the early 20^th ^century, the move of place of birth from home to hospital from the 1970's onwards and findings from large national surveys that this is the area of maternity care that women find the least satisfactory [2,5,15]. A survey of women's experiences of postnatal care published in 2000 [31] was the first in the UK to specifically focus on this aspect of maternity care from the women's perspective, and considered community as well as hospital-based care. Women who had given birth within the previous year were accessed through the member's journal of the National Childbirth Trust (NCT) and an on-line internet site for parents, with 960 completed surveys received. During the first three days following the birth, when women were most likely to be on the postnatal ward, around half of this self-selected group of women reported that they received the information, advice, care and emotional support that they needed. One in ten reported they received very little or no information and a quarter suggested they received no emotional support. The NCT recently published the results of a postnatal care survey undertaken during 2009/2010 involving 1260 first time mothers, most of whom were NCT members (95%) [32]. The authors concluded that there had been limited improvement in postnatal care over the last decade, with some evidence of a decline in meeting woman centred quality-standards, despite greater investment in the NHS and publication of NICE postnatal care guidance.

The experiences of women in the current study are reflected in studies from other countries, where a similar system of hospital-based birth care is the prevalent model, most notably Sweden and Australia [6,8,9,33]. Of note is that several of these studies were also surveys which questioned women several weeks or months following the birth. Nevertheless, the consistency of findings in our study and the earlier studies highlights the continuing low priority accorded to the crucial hours and days following birth. This could potentially negate a good birth experience and a positive start to parenting for the mother, her partner and the infant. The themes we derived from the interview data confirm findings of other researchers [8,33], suggesting that despite differences in context, culture, service organisation and delivery across different Western countries, women's views of their needs and expectations following birth are remarkably similar.

With respect to the physical environment of the ward, a number of the ward routines were viewed by the women in the current study as not conducive to them sleeping or resting following birth. Routines reflected those of a 'traditional' acute medical or surgical ward rather than organised to optimise recovery from birth. The early morning waking was problematic for some women as many would have been awake during the night to feed their babies. Similarly the need to conduct non-urgent medical procedures such as removing a urinary catheter at 6 am in the morning was viewed negatively. It would be useful to consider if in-patient postnatal wards need to have such inflexible routines. Rather than placing women at the centre of care, ward routines likely to have been established as part of an acute medical organisation may be more appropriate for the staff than for women. The lack of flexibility in the in-patient environment has been reported elsewhere. Rudman and Waldenstrom [8] reported on women's negative experiences of hospital postnatal care in a prospective longitudinal study of 2783 Swedish-speaking women surveyed in early pregnancy and at two months and one year postpartum. Negative statements about postnatal hospital care included dissatisfaction with the physical environment of the ward, the room temperature being too high, shabby rooms and bad mattresses. Singh and Newburn [31] in their UK survey also reported that women found postnatal wards too hot, too noisy, and rules about visiting on the wards 'problematic'.

The importance of a two hour 'quiet time' each day, when visitors were not allowed onto the wards, was reported as a positive routine by several women in our study. A busy postnatal ward environment, where staff and visitors are coming and going could problematic for some women in terms of rest and recuperation - it also contrasts with the emphasis on the importance of a peaceful birth environment, where great care is taken to protect a woman's privacy and limit those who enter the birth room. Many women are on 'view' for the duration of their in-patient postnatal stay with all inter-actions and conversations with staff and relatives conducted in full sight and sound of the other women and their visitors. Interestingly, in our interviews lack of privacy did not appear to be problematic, indeed some women reported that they preferred to be in a room with others and they did not want to be alone; the issue is protecting time to enable women to rest. Access to the postnatal wards by visitors is an on-going area of contention given the need to promote maternal well-being but also acknowledge birth as a celebratory social event and important transition for the woman and her partner. Restricted visiting has been viewed as detrimental for the partner as it could interrupt their interactions with their infant, and influence their experience and satisfaction with postnatal care [34,35]. Conversely 'open' visiting may limit a woman's opportunity for rest and opportunities to speak to a midwife if she has any particular concerns. Staff may not wish to interrupt a woman if she is perceived as 'busy' with her visitors.

Previous studies have highlighted the negative impact that staff attitudes and poor communication have on women [6,8,32]. Women in our study for the most part, described staff very positively, although a small minority reported that a member of staff had spoken to them in a manner which they thought was inappropriate. Brown et al [6] found that the greatest effects on women's overall rating of their in-patient care were based on women's inter-action with their caregivers, including how sensitive and understanding caregivers were, how rushed they seemed and if advice and support were offered. There is also evidence that the attitude of postnatal ward staff towards fathers is problematic, with positive encouragement around the transition to parenthood lacking [8], although this was not a finding of our study. The women in our study valued staff being friendly and helpful which concurs with the recommendations of previous studies that there should be a greater focus on the communication and listening skills of postnatal staff [6,35]. Concerns about midwifery staffing levels in the NHS is adding to pressures on the postnatal wards [32,36], resulting in women feeling guilty about calling staff as illustrated in our study. This also emphasises the perception commonly reported [3] that women view staff on the wards as too busy and further calls into question for whose benefit the current organisation and delivery of in-patient postnatal care is intended.

Women's views of support for breastfeeding also highlighted the importance of staff attitudes. The level of support women received on the postnatal ward for breastfeeding prompted mixed responses. It was disappointing that some women found staff unhelpful which left them with the impression that they did not want to support breastfeeding, but also encouraging that others had a very positive experience of staff support. There was particular praise for the nursery nurses, which suggests that there is an important role for all members of postnatal ward teams to play in support for breastfeeding. The crux, as evidenced in the responses reported here, was how individual members of the postnatal team interacted with women, which is likely to be influenced as much by workload pressures, as their knowledge and awareness of how to inter-act with women who wish to breastfeed. Dykes [37] in an ethnographic study of encounters between midwives and breastfeeding women in postnatal wards in England reported that the needs of breastfeeding women for informational and practical support, as well as for emotional, and esteem needs were largely unmet as a result of lack of midwifery time, no established relationship with the women, and the structural constraints of a medicalised organisation. Dykes [37] also identified that it was not just organisational culture which determined the nature of interactions between midwives and women. It was also the encounter with individual midwives who had a range of approaches to supporting breastfeeding women, some facilitative and some didactic, a finding which our data supports. Strategies to increase breastfeeding, such as the WHO Baby Friendly Hospital Initiative [29] include the need to train all health staff in skills to implement the breast-feeding policy. Consideration should also be given to ensuring staff are aware that the way they speak to women about breastfeeding and other aspects of postnatal care could have a considerable and lasting impact on women's experiences.

With respect to unmet information needs, NICE [21] recommends women and their partners receive relevant and timely advice tailored to their individual needs commencing with transfer to the postnatal ward. Women in this study valued information to support their transfer home but also wanted information on their admission to the ward about ward routines and the ward layout. Frequent reference was made to the requirement for advice on practical care of their baby such as bathing and nappy changing. During the last decade the duration of in-patient stay has declined considerably in the NHS [15]. Early discharge home does not appear to have an effect on maternal health or breastfeeding outcomes [38], but has resulted in a system of care where staff have to complete all admission and discharge processes within severe time constraints, leaving little time to implement the care women *perceive *they need and *expect *they will receive from their in-patient stay such as advice on how to care for their babies.

## Study limitations

There were several limitations of the study. As data were based on replies from 20 women, generalisability across the UK could not be claimed, although themes identified were very similar to those reported elsewhere [6-10]. In common with studies which have considered views of service users in the general population [38] women may have felt reluctant to complain about their care, especially when still in hospital. Whilst views may be more positive having been influenced by the 'halo' effect of having just given birth, we have captured data on women's views 'as it happened' and were not relying on their recall weeks or months after the event. It is acknowledged that there may be some bias given that few women had a spontaneous vaginal birth. Most of the women in our sample had experienced a caesarean birth, were aged over 30 and had given birth to their first baby. It is possible that their needs and views were very different to those of, for example, a younger multiparous woman who has had a spontaneous normal birth. Whilst this could reflect that these women were likely to have remained on the wards for a longer period of time and hence were more amenable to being interviewed, it is also likely that these women had more time to consider their experiences and expectations of care.

## Conclusion

Postnatal hospital care in many Western countries is rarely viewed or planned as part of a continuum of planned, effective maternity care for individual women. It remains an over-looked aspect of maternity service delivery despite being the area that is least favourably reported by the women who use the service. Our findings identified that although many women had a good experience on the postnatal wards there was room for improvement in a number of areas including the environment and routines of the postnatal ward, the communication skills of staff, consistency in breastfeeding support and the offering of appropriate and relevant information to support women and their partners on transfer home. The organisation and delivery of in-patient postnatal care is rooted within the culture of a medical organisation designed and organised to take care of people with acute medical illness, which is not appropriate for the majority of women recovering from birth. Innovative changes are required to address this issue, given that there is unlikely to be a significant change in the numbers of women birthing in hospital in the foreseeable future. Current UK policy is focused on safe, high quality care for all [30], and the importance of involving service users in the re-design of a patient-focused healthcare system is acknowledged (40). This is an issue which has to be tackled within NHS maternity services if the quality of in-patient postnatal care is to improve in line with what women expect to receive and achieve policy ambitions.

## Competing interests

The authors declare that they have no competing interests.

## Authors' contributions

DB conceived the study, was lead investigator, participated in its design and data analysis and helped to draft the manuscript. SB participated in the data analysis and coordination of the study and drafted the manuscript. VR collected the data and contributed to the manuscript. AW and JW contributed to the design of the study and the manuscript. All authors read and approved the final manuscript.

## Pre-publication history

The pre-publication history for this paper can be accessed here:

http://www.biomedcentral.com/1471-2393/10/70/prepub
